# The Potential of Zero Charge and the Electrochemical Interface Structure of Cu(111) in Alkaline Solutions

**DOI:** 10.1021/acs.jpcc.0c09289

**Published:** 2021-03-01

**Authors:** Andrea Auer, Xing Ding, Aliaksandr S. Bandarenka, Julia Kunze-Liebhäuser

**Affiliations:** † Institute of Physical Chemistry, University Innsbruck, Innrain 52c, Innsbruck, 6020, Austria; ‡ Physics of Energy Conversion and Storage (ECS), Physics Department, Technical University of Munich, James-Franck-Straße 1, 85748 Garching, Germany; § Catalysis Research Center TUM, Ernst-Otto-Fischer-Straße 1, 85748 Garching, Germany

## Abstract

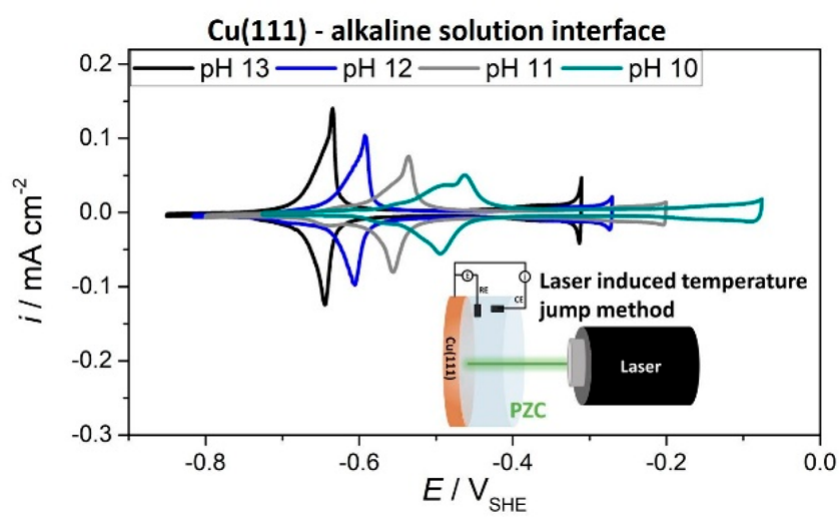

Copper (Cu) is a unique electrocatalyst, which is able to efficiently oxidize CO at very low overpotentials and reduce CO_2_ to valuable fuels with reasonable Faradaic efficiencies. Yet, knowledge of its electrochemical properties at the solid/liquid interface is still scarce. Here, we present the first two-stranded correlation of the potential of zero free charge (pzfc) of Cu(111) in alkaline electrolyte at different pH values through application of nanosecond laser pulses and the corresponding interfacial structure changes by *in situ* electrochemical scanning tunneling microscopy imaging. The pzfc of Cu(111) at pH 13 is identified at −0.73 V_SHE_ in the apparent double layer region, prior to the onset of hydroxide adsorption. It shifts by (88 ± 4) mV to more positive potentials per decreasing pH unit. At the pzfc, Cu(111) shows structural dynamics at both pH 13 and pH 11, which can be understood as the onset of surface restructuring. At higher potentials, full reconstruction and electric field dependent OH adsorption occurs, which causes a remarkable decrease in the atomic density of the first Cu layer. The expansion of the Cu–Cu distance to 0.3 nm generates a hexagonal Moiré pattern, on which the adsorbed OH forms a commensurate (1 × 2) adlayer structure with a steady state coverage of 0.5 monolayers at pH 13. Our experimental findings shed light on the true charge distribution and its interrelation with the atomic structure of the electrochemical interface of Cu.

## 1 Introduction

In recent years, fundamental understanding of the electrochemical interface has been significantly improved, and this can help to further promote the development of alternative systems for clean energy provision. These advances are a prerequisite for the design of efficient electrode materials and interfaces, which allow the transformation of chemical into electric energy or *vice versa* and the synthesis of valuable chemicals.^1^ Understanding the electrochemical Cu/liquid interface is of special interest, since Cu has been shown to not only efficiently electro-oxidize CO in alkaline media at low overpotentials through self-activation by the formation of high-energy undercoordinated Cu adatom structures, as we could show recently,^2^ but also reduce CO_2_ and CO to valuable hydrocarbons and alcohols with reasonable Faradaic efficiencies.^3^ This means that Cu is particularly intriguing in terms of its ability to act as a bidirectional electrocatalyst.

One of the most central characteristics of the solid/liquid interface is the potential of zero charge (pzc), where no excess charge prevails at the electrode,^4^ and the disorder of the interfacial water is close to a maximum.^5,6^ The pzc influences all electrosorption properties and is thus significantly relevant for electrocatalysis in general. In the case of specific adsorption at nonideally polarizable electrodes, which adsorb hydrogen and hydroxide (OH) species, such as Pt^7^ and Cu,^8,9^ it is important to distinguish between total and free charge density.^4,6,10,11^ The total charge includes both the surface charge, i.e. the true electronic charge on the electrode, and the fraction of charge, which is involved in the adsorption process and which consequently has crossed the interface by being transferred, e.g. from the metal to the solution side.^12^ The free charge only reflects the true excess charge on the electrode surface and is therefore mainly responsible for the electric field at the interface. The potential where the total charge density vanishes is called potential of zero total charge (pztc), and the potential where the true surface excess charge density becomes zero is the potential of zero free charge (pzfc).^11^ The strength of the interfacial electric field, as obtained by the difference between the pzfc and the actual applied electrode potential, heavily affects the electrochemical reactivity. Far away from the pzfc, at high electric fields, a rigid water layer exists very close to the electrode surface, which kinetically hinders charge transfer. Close to the pzfc, the water dipoles have a much higher degree of freedom and can more easily reorient upon charge transfer. Therefore, the activity and rate of any charge transfer reaction critically depends on the position of the pzfc, which dictates the strength of the electric field, because this determines how easily the solvent can accommodate for charge migration.^13^

Determination of the pzfc can be experimentally achieved by applying the so-called laser-induced temperature jump method.^5,6,14−16^ In principle, there are two different measurement procedures: (i) coulostatic laser-induced potential transients^6,15,17^ and (ii) potentiostatic laser-induced current transients (LICT).^14,16,18^ Both rely on nanosecond laser pulses inducing a sudden temperature increase at the electrified interface, which causes a distortion of the interface structure. This temperature change as well as the response of the electrode take place at a very short time scale in the submicrosecond range.^14^ This allows the separation of purely capacitive processes from Faradaic processes and thus correlation of the pzt with the pzfc, because specific adsorption definitely occurs at a slower rate.^5,19,20^ With method (i), a change is induced at the open circuit potential, which, depending on its sign, is indicative of the orientation of the water dipoles. Method (ii) has been applied in this work: it leads to a certain distortion of the electrified double layer upon heating and is based on subsequent detection of the charge during reorganization of the double layer upon rapid cooling. If the electrode is negatively charged, the nanosecond laser pulse results in negative current transients, and if it is positively charged, positive current transients are measured. The potential of zero transient (pzt) is located at the potentials where the transients change their sign. It is also referred to as potential of maximum entropy (pme) in the case of method (i).

The laser-induced temperature jump method has been successfully employed for the study of interfacial fundamentals of low-index Pt,^6,19−22^ Ir,^5^ and Au^14,15^ single crystals as well as for highly stepped Pt electrocatalysts.^23^ More recently, the electric field strength and the order of interfacial water could be correlated with the activity toward the hydrogen evolution reaction (HER) of Ni(OH)_2_ modified Pt electrodes.^13,24^ Numerous previous studies also addressed the pH dependence of the interfacial parameters, especially for Pt group metals.^5,19,20,22,23^ Despite the importance of copper in electrocatalysis under alkaline conditions, the actual value of the pzc at the Cu/electrolyte interface under these conditions has only been reported very recently.^25^ In this study, coulostatic laser-induced potential transients have been employed for the examination of the potentials of maximum entropy (pme) of Cu(111) and Cu(100) at pH 13.^25^ It is known that both pH and adsorption processes strongly influence the interfacial electrochemical properties of metal electrodes,^5,10,19^ i.e. their pzc, and their atomic structures.^26^ Electrochemical scanning tunneling microscopy (EC-STM) is a powerful tool to visualize the adsorbate- and potential-induced structural changes at the interface *in situ* under perfect electrochemical control. In the case of Cu(*hkl*), electrochemical scanning probe microscopy has significantly contributed to the understanding of important interfacial processes such as SO_4_^2–^^27,28^ and OH^–^ electrosorption.^9,29−31^ OH adsorption on Cu(111) is known to proceed together with a surface reconstruction where the density of the first Cu layer decreases and excess Cu is ejected to the top of the terraces in the form of small clusters.^9,29^ Previous studies regarding the OH adsorption on Cu(111) electrodes in 0.1 M NaOH found an ordered hexagonal OH adsorbate layer with a structural parameter of (0.6 ± 0.2) nm and one adsorbate per unit cell, corresponding to a low coverage of only ∼0.2 monolayers (ML) of adsorbed OH species with respect to the unreconstructed Cu(111).^9,29^ Based on the knowledge of the adsorbate lattice parameter, it was assumed that the outermost Cu layer reconstructs to adopt the Cu plane structure in Cu_2_O (111) with a Cu–Cu distance of 0.3 nm, on which the OH adsorbate forms a (2 × 2) superstructure.^9,29^ Correlations of copper’s activity toward catalytic processes, such as the hydrogen evolution,^32,33^ CO oxidation,^2^ and CO_(2)_ reduction,^34−36^ with interfacial structures has also been shown.

In this work, we provide for the first time a pH-dependent pzfc value for Cu(111) in alkaline solutions using LICT. We combine these studies with *in situ* EC-STM investigations of the electrified Cu(111)/liquid interface structure and dynamics close to the pzfc at two different pH values. This combination allows us to correlate the sign of the true charge at the interface, i.e. the position of the pzfc, with the morphological and structural evolution of the Cu(111) surface. While knowledge of the pzfc in alkaline media is highly relevant on its own, our combined *in situ* study unambiguously provides evidence for a shift of the pzfc with pH. We demonstrate that the pzfc is located at the onset of the Cu(111) reconstruction. This clear correlation emphasizes the role of the free charge on the atomic structure of Cu surfaces. We also provide new structural information on the OH adsorption on Cu(111) at pH 13. Our double-stranded approach helps to generally clarify the role of the electric field on the Cu(111) reconstruction and on the adsorption of electrolyte species. This fundamental understanding is an essential step toward unraveling the complex electrochemical behavior and electrocatalytic activity of Cu(111) electrodes.

## 2 Experimental Section

### 2.1 Chemicals and Materials

The electrolyte solutions were prepared from NaOH (99.99%, trace metal basis, SigmaAldrich), HClO_4_ (99.99%, Suprapur, Merck), and ultrapure water (Milli-Q purification system, >18 MΩ cm, Merck). Ar (Messer, 5.0) was used for deaeration of the electrolytes. Cu(111) crystals (Mateck, Jülich) were mechanically polished with diamond paste (ESCIL) down to 0.25 μm, electropolished in 60% H_3_PO_4_ (85% EMSURE, Merck) at 1.8 V versus a Cu counter electrode and annealed in a homemade horizontal tube furnace under H_2_ flow prior to each experiment. The crystals can be removed while maintaining the H_2_ atmosphere, which allows a direct transfer to an Ar-filled (Messer, 5.0) glovebox (Mbraun MB 200 MOD glovebox) to perform the electrochemical scanning tunneling experiments without any traces of oxygen from air.

### 2.2 Electrochemical and Laser-Induced Current Transient Measurements

All cyclic voltammetry measurements were performed in a standard three-electrode cell configuration, where the electrolyte was filled in a Teflon beaker to avoid any contaminations from glassware. The use of glassware was found to significantly alter the peak shape of the OH adsorption on Cu(111);^37^ we found however no changes in the measured charge obtained from integration of the current. All cell parts were cleaned in KMnO_4_, piranha solution (H_2_SO_4_ and H_2_O_2_), and ultrapure water prior to the experiments. A carbon rod counter electrode and either a polytetrafluoroethylene (PTFE) bound activated carbon quasi-reference (AC-QRE) or a Ag/AgCl (saturated KCl) reference electrode were used for the electrochemical experiments, and the reference potentials were converted to the standard hydrogen electrode (SHE) scale. All experiments were carried out in completely deaerated, Ar-purged solution using a Biologic VSP 300 (France) potentiostat. Laser-induced current transient (LICT) measurements were carried out in a standard, three-electrode glass cell, which is unavoidable due to the specific setup; unpublished results show no effect of the glassware on the position of the pzfc. A Quanta-Ray INDI pulsed Nd:YAG laser with a wavelength of 532 nm was used for these measurements. Laser pulses with a duration of 5–8 ns (repetition rate of the laser pulse 10 Hz) and an energy of 67 mJ cm^–2^ were applied to the Cu(111) crystals with an area of 0.50 cm^2^. The spot size of the laser was adjusted to a diameter of 9 mm. All LICT measurements were performed potentiostatically within the potential range from −1.0 to −0.36 V_SHE_, which is well below the oxidation onset of Cu(111),^29,33^ with a step size of 20 mV. A detailed description of the LICT setup is given in refs (16 and 18).

### 2.3 Electrochemical Scanning Tunneling Microscopy

Electrochemical scanning tunneling microscopy (EC-STM) experiments were performed with a Keysight 5500 scanning probe microscope inside an Ar-filled glovebox to avoid any oxygen contamination. A home-built polychlorotrifluoroethylene (PCTFE) EC-STM cell was employed with polytetrafluoroethylene (PTFE) bound activated carbon quasi-reference and counter electrodes, which is described in detail in ref (38). Electrochemical etching of a tungsten wire was employed to prepare the STM tips, which were subsequently coated with Apiezon wax. The Cu(111) electrode was immersed at open circuit potential, and the formed native copper oxide monolayer was reduced prior to every experiment. The metallic surface was systematically imaged to ensure high quality and large terrace sizes. Representative STM images of the metallic Cu(111) surface are shown in Figure S1 in the Supporting Information (SI). Data representation of the STM images was performed with Gwyddion.^39^

## 3 Results and Discussion

**Figure 1:**
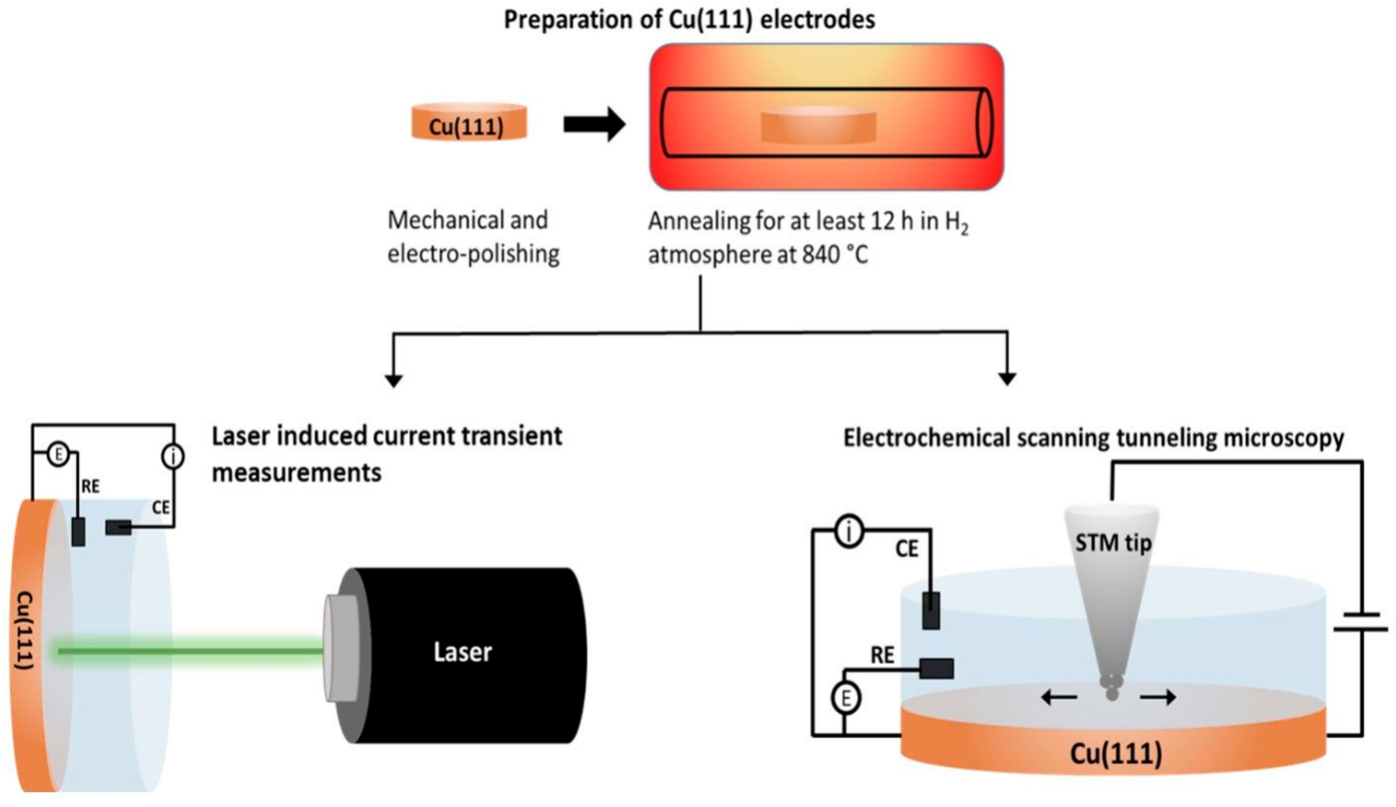
Scheme of preparation and characterization of the electrified Cu(111)/liquid interface. The Cu(111) crystals are reproducibly prepared by mechanical and electro-polishing and subsequent treatment in a H_2_ atmosphere at 840 °C for over 12 h. The interdependence of the interface parameters and the structure is investigated *in situ* by laser-induced current transient (LICT) and electrochemical scanning tunneling microscopy (EC-STM) studies.

To determine the interdependence of thermodynamic interface parameters and the atomic structure at different pH values in alkaline media, Cu(111) single crystals were investigated via a two-pronged approach, as illustrated in Figure 1. After careful preparation of the Cu electrodes (described in detail in the Experimental Section), they were transferred either to the laser-induced current transient (LICT) setup to determine the position of the potential of zero free charge (pzfc) or to the EC-STM setup to visualize the structures formed at the solid/liquid interface at the potentials of interest.

**Figure 2:**
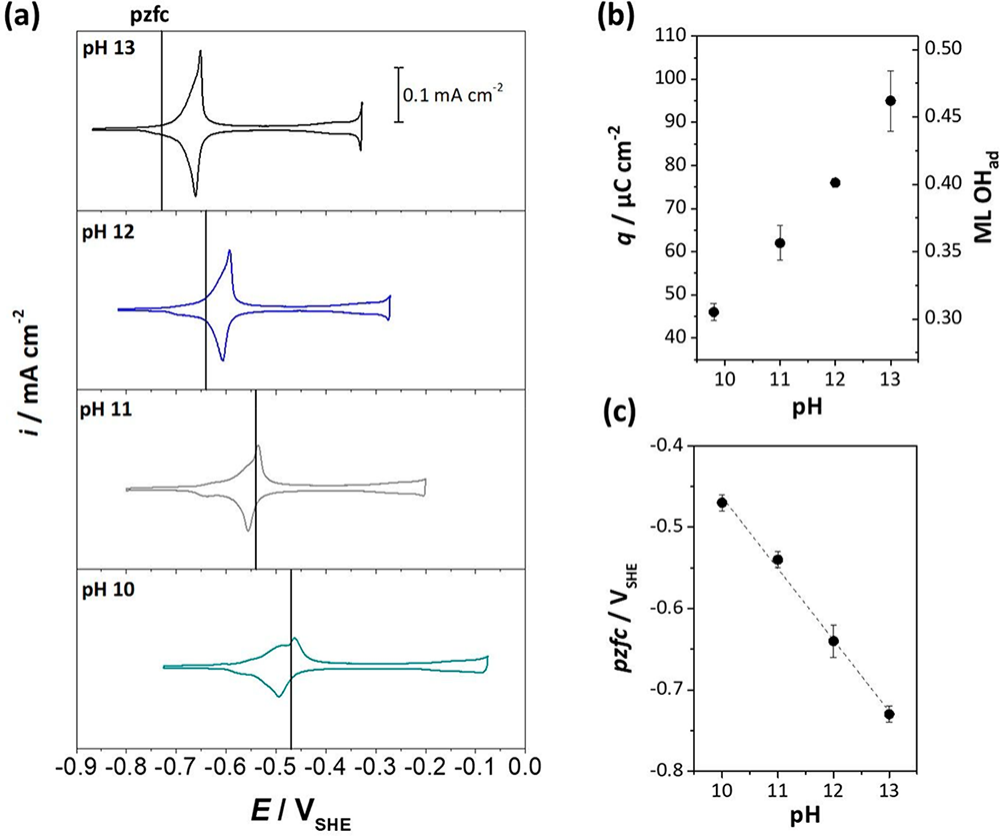
Interdependence of the potential of zero free charge (pzfc) and current versus potential cycles. (a) Cyclic voltammograms (CVs) of Cu(111) in 0.1 M NaClO_4_ at different pH values showing the electrochemical behavior in the potential range between −0.9 V_SHE_ and 0.0 V_SHE_ (scan rate: 50 mV s^–1^). The observed peak pairs correspond to the adsorption and desorption of OH^–^ ions from the alkaline solutions. The vertical lines mark the positions of the pzfc. Dependence of (b) the estimated charge, i.e. the coverage with apparent OH monolayers, and (c) the pzfc on the pH. The error bar in (b) is determined through averaging of the charges of at least 3 CVs; the error bars in (c) correspond to the widths of the *x*-axis intercepts determined from Figure 3a–d.

**Figure 3:**
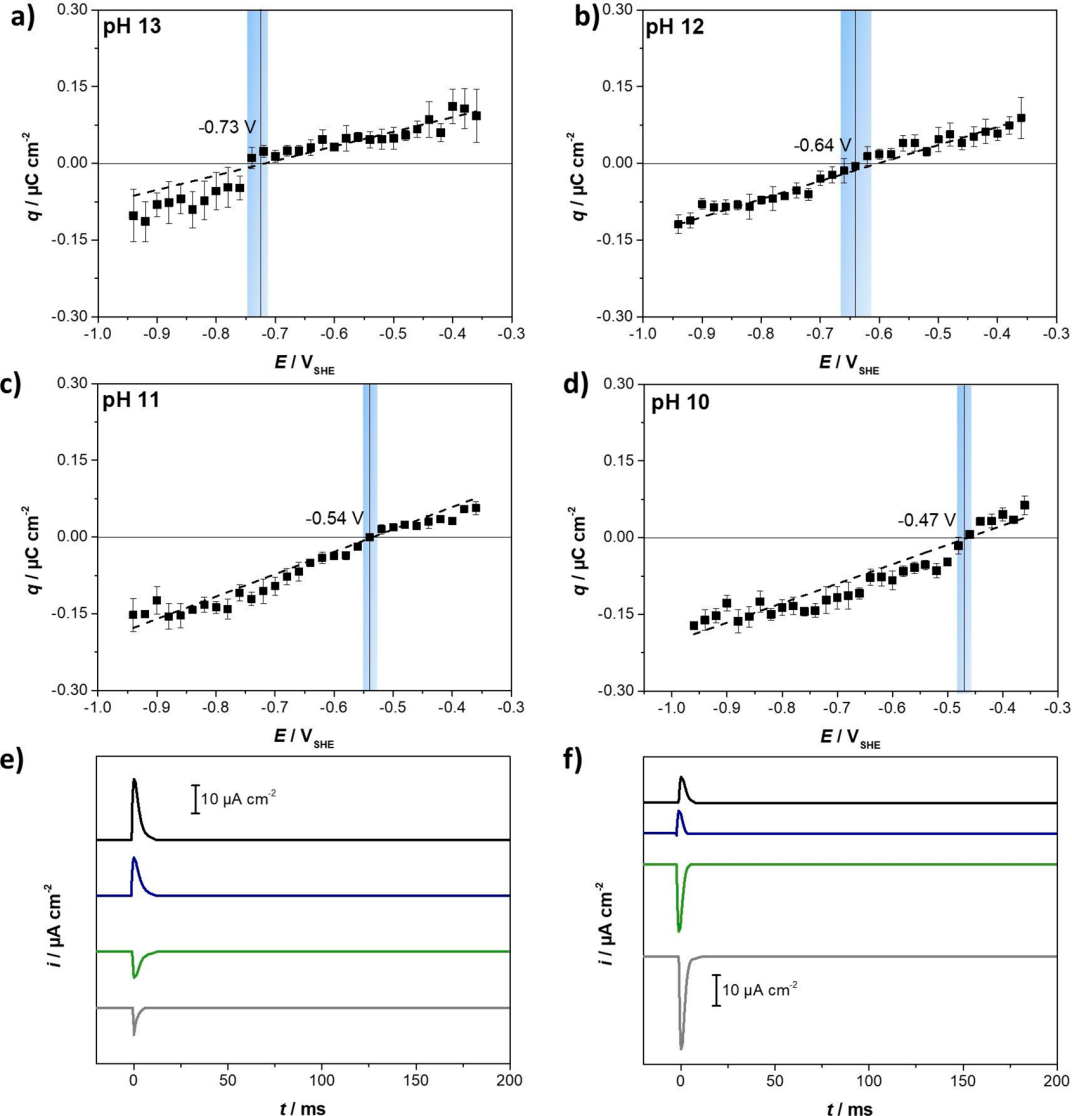
pH dependence of the pzfc at Cu(111). Calculated charge densities *q* from integration of the current transients vs *E* in 0.5 M NaClO_4_ at (a) pH 13, (b) pH 12, (c) pH 11, and (d) pH 10. Examples of the recorded transients at selected potentials after background subtraction are shown for pH 13 (e) and pH 11 (f); selected semilogarithmic plots are shown in Figure S5 in the Supporting Information.

Cyclic voltammograms (CVs) combined with the corresponding LICT results constitute the fundament of our investigation, because they provide a macroscopic overview of the potential range of interest of Cu(111) in NaClO_4_ solution for four pH values in the alkaline regime (Figure 2). The voltammetric profiles (Figure 2a) of the Cu electrode show the characteristic peak pair related to the adsorption and desorption of OH with maxima at around −0.65 V_SHE_ (0.12 V_RHE_) for pH 13, which is in perfect agreement with the literature.^25,37,40^ The OH adsorption peak shows ideal Nernstian behavior with a shift of approximately 59 mV/decade in the investigated alkaline pH range (pH 13–10). It clearly exhibits two regions with different slopes in the current response, which becomes more pronounced at lower pH values. At pH 10, there are clearly two features which can be distinguished. This indicates different adsorption sites, presumably steps at lower potentials and terraces at higher potentials. The current profile of the first feature, attributed to step edge adsorption, appears less steep than the second one. With lower pH, i.e. lower OH^–^ concentration, it flattens further. The sharpness and reversibility of the feature at higher potentials, which might be due to a phase transition of the OH adsorbate, e.g. to an ordered adlayer, is largely conserved for pH 13 and 12 and flattens at pH 11 and 10. The estimated charge from integration of the current density decreases linearly with decreasing pH, exhibiting apparent OH coverages of 0.46 monolayers (ML) at pH 13 and 0.22 ML at pH 10 (see Figure 2b). We must note that, in contrast to consistent CVs of single crystalline Au and Pt, the voltammetric response of Cu in alkaline solution shows small discrepancies over the literature, which is due to the influence of surface pretreatment, applied potential range,^41^ and effect of dissolved glass ware.^40^ Nevertheless, the CV at pH 13 in Figure 2a clearly does not show any additional feature due to the presence of perchlorate ions in the alkaline solution (see the CV of Cu(111) measured in 0.1 M NaOH shown in Figure S2 for comparison), which means that no visible specific adsorption of ClO_4_^–^, i.e. a charge transfer reaction, occurs. The pzt values for Cu(111), i.e. the pzfc, where no excess free charge resides on the metal, are measured at −0.73 V_SHE_, −0.64 V_SHE_, −0.54 V_SHE_, and −0.47 V_SHE_ for pH 13, 12, 11, and 10, respectively (see vertical lines in Figure 2a and Figure 3), which corresponds to a shift of (88 ± 4) mV per pH unit on the SHE scale or approximately 29 mV/dec on the RHE scale (see Figure 2c). At pH 13, the pzt is located within the apparent double layer region, before the onset of the OH adsorption in good agreement with the pme of −0.71 V_SHE_ for Cu(111), that was very recently reported in ref (25). It continuously shifts toward higher potentials for lower pH values and is therefore located within the OH adsorption region for both pH 11 and 10.

Figure 3 depicts the calculated charge densities *q* from integration of the current transients of Cu(111) at the four different pH values as functions of the electrode potential *E*. The corresponding maximum current values are shown in Figure S3. Mean values and standard deviations of the charges result from averaging six (pH 13 and 12) or three (pH 11 and 10) transient data sets (see Figure 3a–d). The *x*-intercepts are determined through linear fits of the *q* versus *E* plots. This linear behavior is expected for an equivalent circuit consisting of the solution resistance and the double layer capacitance in series and is, therefore, representative for the double layer region of the given Cu(111)/liquid interface. However, it has to be noted that the double layer charge is only partially measured with LICT.^42^ Remarkably, the slopes of the *q* versus *E* plots are all in the same capacitance range between 0.3 and 0.4 μF cm^–2^, independent of the pH value, and a linear charge increase is also observed at potentials more positive than the OH adsorption regions in all cases. This strongly indicates that the specific OH adsorption charge does not contribute to the laser-induced current transients.

The original LICT data are shown as 3D plots in Figure S4. All recorded current transients in the whole pseudocapacitive potential range show a rather monotonic decay after the sharp increase due to the laser pulse (Figure 3e,f). Semilogarithmic plots of two representative current transients are depicted in Figure S5. The mostly linear decay of the transients implies that the dominating contribution to the responses is solvent reorganization,^5,25^ i.e. that no specific adsorption process is measured. Bipolar or nonmonotonous responses would clearly indicate an overlap of different processes with unequal rates, such as double-layer restructuring and specific adsorption of ions. Such nonmonotonous profiles have been previously reported for highly acidic or alkaline conditions and are due to fast adsorption processes.^19,20^ In the present case, the recorded decay is influenced by the large cell constant of 160–250 Ω, as it is usually found in LICT measurements, which makes kinetic studies of the relaxation process in the microsecond time-scale difficult, and which limits the accuracy for the detection of different time constants. However, laser-induced current transients are well deployable for the determination of the pzfc if specific adsorption contributions can be excluded. Here, the absence of the contribution of specific adsorption can be based on the linear *q* versus *E* trends in Figure 3 that show the same slopes for all four pH values.

A similar pH dependence of the pzfc, determined with the temperature jump method, has been reported for the case of Pt(111) in alkaline solution, where a positive pzt shift with decreasing pH is found resulting in pzfc values lying within the OH adsorption regime.^20^ This is in contrast to acidic or neutral media, where the pzfc of Pt electrodes was found to be constant with pH on the SHE scale.^22^ A comparable shift of the pzt by 30 mV/dec on the RHE scale has been reported for Ir(111) electrodes in solutions ranging from pH 5 to 1.^5^ The pH dependence of the pzfc of metals that adsorb hydrogen and OH species, e.g. Pt group metals, has been described through theoretical thermodynamic analysis with the following equation:^10^
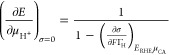
1where *E* is the electrode potential measured versus a constant reference electrode,^10^ μ_H^+^_ and *μ*_CA_ are the chemical potentials of the H^+^ and the salt (CA: cation, anion) ions, σ is the free charge density, *FΓ*_H_ is the thermodynamic excess charge of adsorbed hydrogen, and *F* the Faraday constant. According to this equation, the pzfc is independent of the pH, when it lies within the double layer region and . If the pzfc lies within the hydrogen region, there are several cases: (i) The adsorption does not involve any change in the free charge, which leads to  and a shift of −59 mV/dec with increasing pH. (ii) The hydrogen adsorption has an effect of  on the free charge, and the potential shift toward negative potentials is even greater than that for the potential of the RHE.^10^ It therefore seems reasonable that an analogous expression exists for the dependence of the free charge on the excess charge of adsorbed OH, FΓ_OH_, which explains the observed shift of the pzfc with pH for Cu(111) in highly alkaline solution. This influence of adsorbates on the free charge is expected to be very similar to an adsorbate induced change in the work function of a metal.^43^

**Figure 4:**
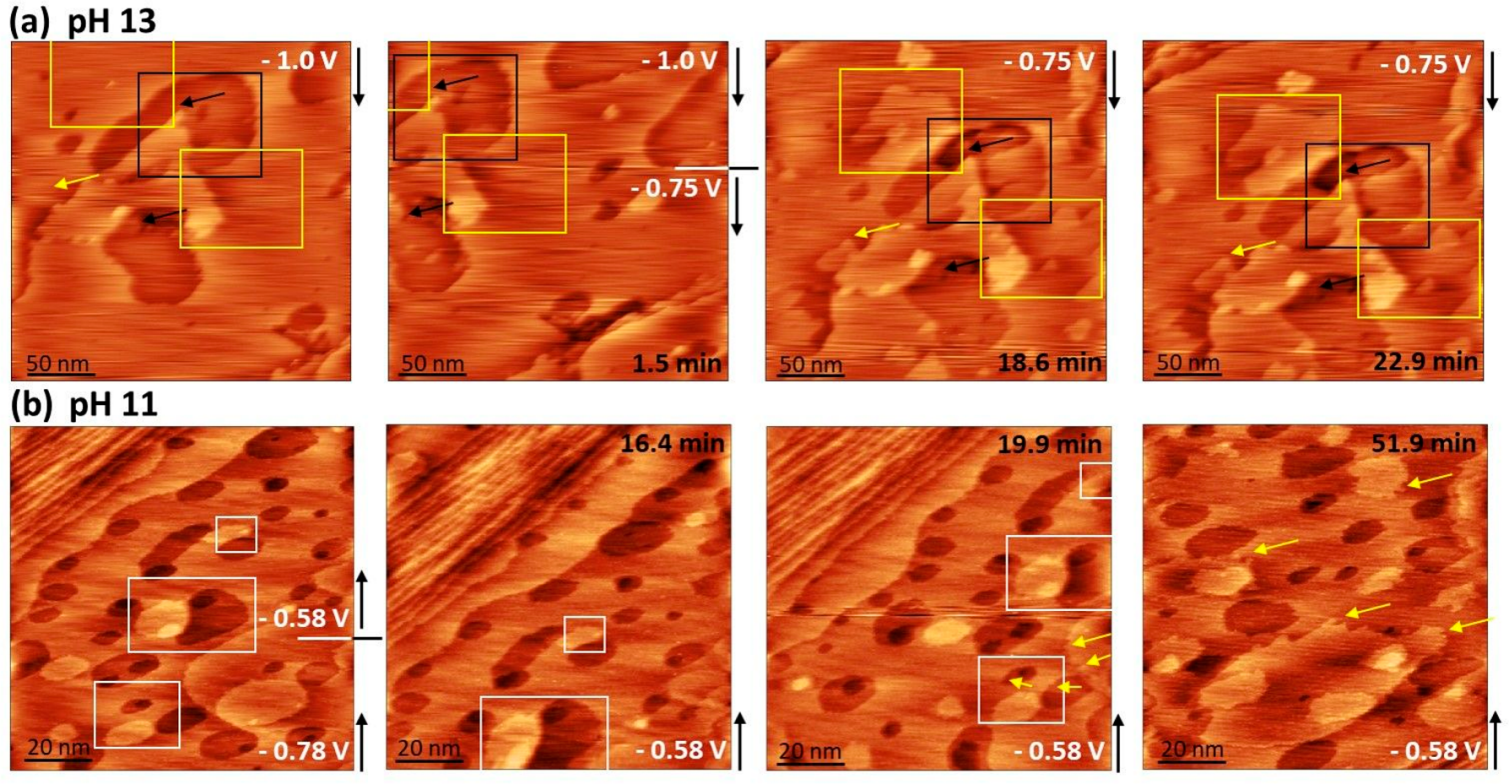
EC-STM imaging to visualize structural and morphological changes at the pzfc. Time- and potential-dependent sequences of EC-STM images at pH 13 (a) and pH 11 (b). The morphology of the surface undergoes gradual changes, which are highlighted with black (vacancy island formation) and yellow (island formation) squares and arrows in (a), marking the position of identical spots on the surface that slightly move due to a drift in the images. The white boxes in (b) mark identical spots, while yellow arrows point to morphologically changed step edges. (a) Size = (250 × 250) nm^2^. (b) Size = (100 × 100) nm^2^, *I*_tip_ = 1 nA, *E*_tip_ = −0.40 V_SHE_.

In order to clarify the interdependence of the reconstruction, the OH adsorption process and the free excess charge at the metal, as determined by the position of the pzfc, *in situ* EC-STM imaging was employed. Due to the observed pzfc shift toward higher potentials with lower pH, and its apparent overlap with the OH adsorption regime (see Figure 2), possible charge and/or adsorption induced interface changes at both pH 13 and pH 11 are investigated. Figure 4 shows EC-STM image sequences during potential steps from the apparent double layer region to the pzfc at pH 13 (Figure 4a) and pH 11 (Figure 4b). At both pH 13 and 11, the metallic Cu(111) surface is first imaged at sufficiently negative potentials *E* < pzfc. Both surfaces are characterized by terraces and (vacancy) islands mostly separated by monatomic steps. After the potential step to the pzfc at pH 13 (−0.75 V_SHE_), small changes of the surface morphology are observed within 15 min (Figure 4a), After prolonged time at the pzfc (∼23 min), substantial (vacancy) island growth and vanishing is visible. Black and yellow boxes and arrows in Figure 4a mark identical positions in the images where vacancy islands or islands form. The observed changes must result from a movement of surface atoms and a starting reconstruction of the topmost Cu layer, which is likely due to an adaption of the surface structure to the change of charge. At a slightly more positively charged electrode (at −0.70 V), gradual surface changes are still observed for over 100 min, but they occur more drastically than at the pzfc (Figure S6 in the Supporting Information). At pH 11, the potential step to the pzfc (−0.58 V_SHE_) does also not lead to abrupt morphology or structure changes (Figure 4b). Only step edge roughening, an indication of OH specifically adsorbing at the step edges, is observed after prolonged imaging of >20 min (see Figure 4b, yellow arrows). The drift in Figure 4b does not allow comparison of identical spots (marked with white boxes in Figure 4b) after prolonged cycling, but careful analysis of the step edge appearance clearly shows that roughening occurs. For additional clarity, low resolution images demonstrating the homogeneity of the Cu(111) surface before and after the step edge roughening are depicted in Figure S7. The appearance and disappearance of islands and the roughening of steps observed at both pH 13 and pH 11 within a comparable time period is a clear indication of surface mass transport.

**Figure 5:**
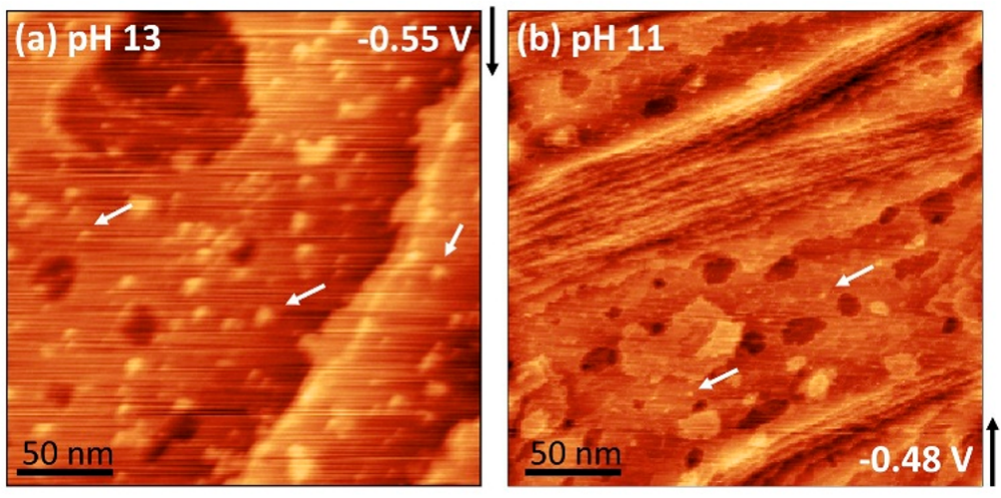
Steady-state surface morphology and structure of Cu(111) after OH adsorption at (a) pH 13 and (b) pH 11. Size = (250 × 250) nm^2^, *I*_tip_ = 1 nA, *E*_tip_ = −0.40 V_SHE_.

At pH 11, the pzfc is positioned where clear Faradaic currents are observed in the CV. Nonetheless, no distinct structural changes are visible on the terraces at this potential. This and the very pronounced step edge roughening supports our hypothesis that the shoulder at more negative potentials, which becomes more pronounced at lower pH values, might be attributed to OH adsorption at step edges. One plausible explanation for our findings is that the pzfc triggers the reconstruction and that the Cu–Cu distance widening is a structural adjustment to the induced positive charge. The role of the sharp feature at more positive potentials in the CVs is elucidated through a potential step beyond this peak and subsequent imaging with EC-STM at pH 13 and 11 (Figure 5). A much higher degree of mass transport and a complete reconstruction of the Cu(111) surface, as manifested by the nucleation and growth of numerous Cu islands,^9^ is observed. After full reconstruction and OH adsorption, the surface morphology and structure are found to be completely static in the time frame of the experiment, which clearly differs from the observations at the pzfc. At both pH 13 and pH 11, small adsorbed Cu clusters are formed due the ejection of excess Cu, which seem to form both round and linear structures that especially reside at the step edges (see Figure 5, marked with arrows). On Cu in alkaline media, this is known to result from the formation of a less dense first Cu layer, where the Cu–Cu distance is increased, and the excess Cu is ejected to the top of the terraces in the form of small, adsorbed clusters.^9,28^

**Figure 6:**
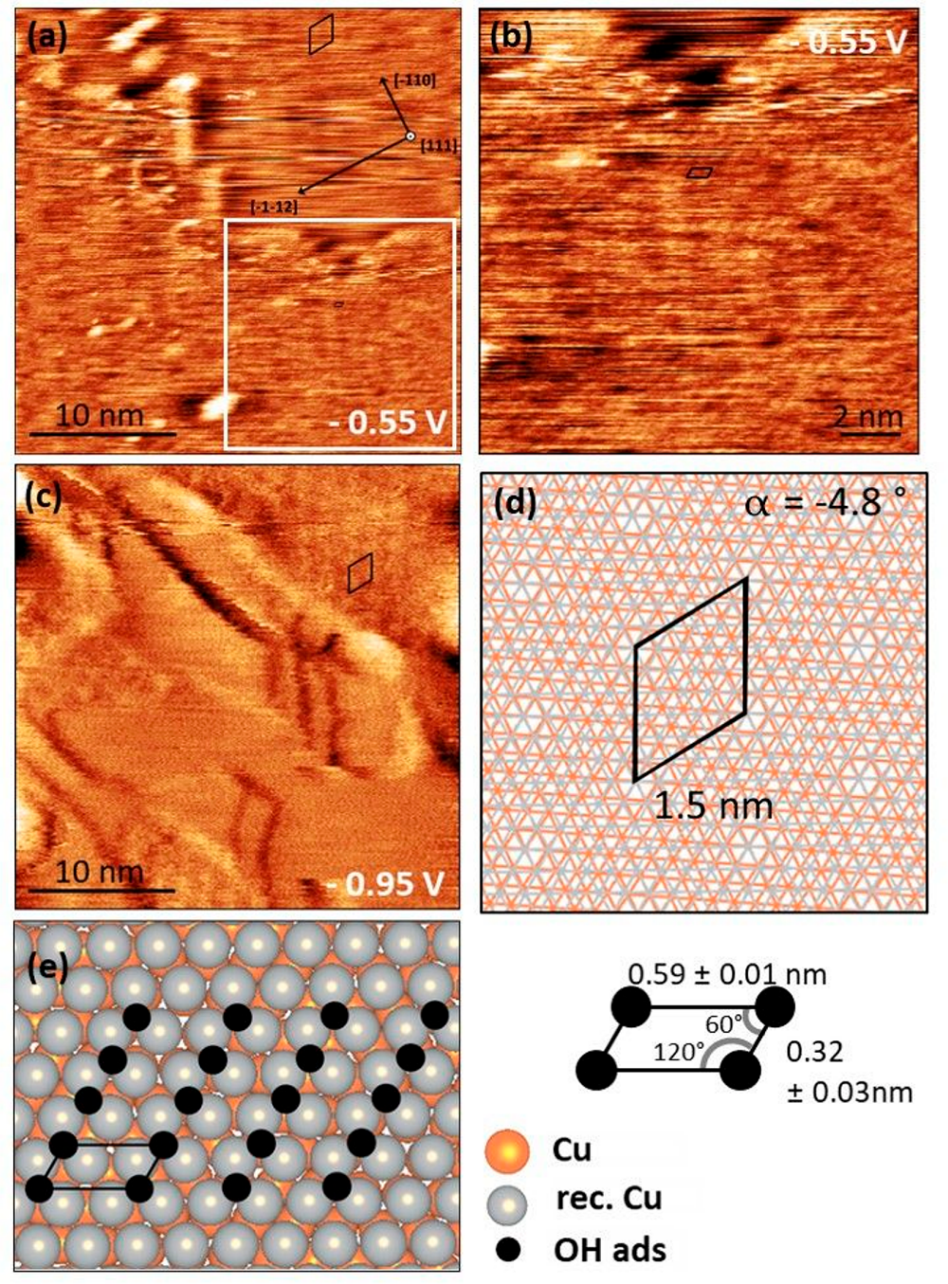
OH-adsorbate structure and its Moiré pattern are visible under exclusion of ambient air at pH 13. ((a) and (b)) High resolution images of Cu(111) at the OH adsorption potential (−0.55 V_SHE_). The zoom marked in (a) is shown in (b). (c) Image of the Moiré structure, which is still partially present even after OH desorption at −0.95 V. Structural models: (d) hexagonal long-range modulation (Moiré pattern), (e) commensurate (1 × 2) superstructure of adsorbed OH on the reconstructed Cu surface.

Previous structure studies of the Cu(111) reconstruction were performed in O_2_ containing alkaline solutions,^9,29^ and it is very likely that due to the high oxygen affinity of Cu, the molecular O_2_ influences the OH adsorption process, e.g. by coadsorption or dissociation at the electrode surface. In the following, we present new structural information on the OH adsorbate layer imaged in completely deaerated alkaline solution at pH 13 (see Figure 6) in an Ar-filled glovebox (see Experimental Section). It is important to note that we could only image this structure at potentials after the sharp feature of the OH adsorption peak. This and the sharp and reversible occurrence of the peak indicates that it originates from a transition from a random to an ordered OH adsorbate layer. High resolution STM images at −0.55 V_SHE_ exhibit both a short-range ordered structure as well as a long-range modulation, which is attributed to a Moiré pattern (Figure 6a and b). A similar coincidence cell was observed upon SO_4_^2–^ adsorption on Cu(111) at positive potentials in dilute acidic electrolyte.^27^ The OH adsorbate induced Moiré pattern is locally persistent after OH desorption, as shown in Figure 6c. The observed hexagonal modulation can be best rationalized by an expansion of the first Cu layer to a Cu–Cu distance of 0.3 nm and a rotation of −4.8° (see Figure 6d). In this model, the Moiré periodicity of 1.5 nm is caused by the lattice mismatch between the first expanded and the second nonexpanded Cu layer. Despite the rather limited quality of the images, due to tunneling in alkaline electrolyte in a glovebox, it can be furthermore assumed, based on the intermolecular distances and angles measured in Figure 6, that the adsorbed OH forms a commensurate (1 × 2) structure on the reconstructed Cu layer. This corresponds to 0.5 ML of adsorbed hydroxyl species with respect to the reconstructed Cu. A possible model including the unit cell is depicted in Figure 6e. It has to be noted that a (2 × 2) OH adsorbate structure formation cannot be excluded if the STM data alone are considered. In the literature, the coverage determined by integration of the current density of the CV usually corresponds to 0.33 ML, which is always related to the unreconstructed Cu(111) layer and thus to a charge of ∼280 μC cm^–2^, which is calculated by assuming the presence of one OH molecule per Cu atom of the unreconstructed surface.^37,44^ The new structural insights provided by the EC-STM images in this work make it possible to correlate the OH adsorbate layer with the reconstructed Cu surface, which corresponds to ∼205 μC cm^–2^, i.e. 1.28 × 10^15^ atoms/cm^2^. These considerations yield ∼0.5 ML OH coverage with respect to the reconstructed surface, both estimated from the CV measurements ((95 ± 7) μC cm^–2^ or (0.46 ± 0.04) ML) and determined from the STM images (see Figure 6). This correlation adds evidence to the proposed OH adlayer structure. A first indication of two different OH adsorption domains is presented in Figure S8 in the Supporting Information. It is noteworthy that, in all the OH adsorption experiments that we conducted under exclusion of air inside the glovebox, the adsorbate structure with its dark appearance initiating to form at the terrace borders, well-known from previous studies,^9,29,45^ could never be observed, while we were able to image it in an EC-STM outside the glovebox under air exposure with the same single crystals (see Figure S9). This clearly indicates that oxygen plays a crucial role for this type of reconstruction. It is very likely that the Moiré pattern has not been visible earlier, due to coadsorption phenomena of oxygen.

The general trend of Cu electrodes to reconstruct dynamically not only upon anion adsorption but also under reaction conditions, e.g., during hydrogen evolution,^32,33^ CO_(2)_ reduction,^34−36^ or CO oxidation,^2^ can be rationalized by its low cohesive energy of 3.5 eV compared to other, more stable metals like Pt (5.84 eV).^46^ Therefore, the restructuring of Cu electrodes, here due to adjustment of the first Cu layer to the induced positive surface charge after the pzfc and subsequent anion adsorption, is an important factor that influences its electrochemical reactivity.

## 4 Conclusion

We have shown that interfacial conditions, such as the potential dependent electrode charge with respect to the pzfc, largely influence the atomic structure of the Cu(111) electrode surface, and *vice versa*, which has essential consequences for electrochemical reactions in which surface adsorbates are involved. This plays a central role in electrocatalysis due to the importance of the catalyst surface properties for intermediate adsorption and activation. For the first time, the pzfc of Cu(111) has been measured as a function of pH in alkaline media and is found to move to more positive potentials by (88 ± 4) mV per decreasing pH unit. While at pH 13 the pzfc lies in the apparent double layer region, prior to the onset of OH adsorption, it continuously shifts toward more positive potentials reaching a position within the OH adsorption regime at lower pH values. *In situ* EC-STM imaging at pH 13 and pH 11 reveal that the Cu(111) surface slowly starts to restructure at the pzfc, where first subtle morphology and structure changes are observed, while no OH adsorbate layer is yet visible on the terraces. This slow change assigned to the onset of the Cu(111) reconstruction is understood as a direct consequence of the change in free excess metal charge that is determined by the position of its pzfc in alkaline solutions. The driving force for the OH adsorption process is then triggered by the increasing positive free charge on the metal with further increasing potential. The adsorption charge directly relates to the strength of the electric field at the interphase. Consequently, the strongest electric field leads to the highest OH adsorption charge at pH 13. The sharp feature in the CV possibly marks the transition to an ordered OH adsorbate layer that can be visualized at sufficiently positive potentials under the conditions applied in this work. At pH 13, high-resolution STM images recorded under oxygen exclusion reveal a (1 × 2) adsorbate layer of OH species on the reconstructed first Cu layer, which expands to a Cu–Cu distance of 0.3 nm upon reconstruction. Due to the mismatch between the lattice parameters of the reconstructed first and the nonreconstructed second Cu layer, a hexagonal Moiré pattern appears. These findings give a realistic picture of the electrochemical Cu(111)/electrolyte interface in an alkaline environment and emphasize the possibility of soft coinage metals, such as Cu, Ag, or Au, which are important in electrocatalysis, to undergo similar reconstructions under electrochemical reaction conditions, which strongly influences their activity.

## Supporting Information Available

Additional EC-STM images for pH 13 and pH 11; CV of Cu(111) in pure NaOH; maximum current values of the transient versus E plot, 3D plots of laser-induced current transients of Cu(111); semilogarithmic plots of current transients; structural model of two rotational adsorption domains (PDF)
