# Supplementary material for: The Potential of Zero Charge and the Electrochemical Interface Structure of Cu(111) in Alkaline Solutions
Source: J Phys Chem C Nanomater Interfaces. 2021 Mar 1;125(9):5020–8. doi: 10.1021/acs.jpcc.0c09289 (PMC8016203; doi:10.1021/acs.jpcc.0c09289)
Supplement: Supplementary file 1 — jp0c09289_si_001.pdf [file jp0c09289_si_001.pdf]

Supporting Information:

The Potential of Zero Charge and the Electrochemical Interface Structure of Cu(111) in Alkaline Solutions

Andrea Auer,[†#] Xing Ding,[‡#] Aliaksandr S. Bandarenka[‡§*] and Julia Kunze-Liebhäuser[†*]

[†] *Institute of Physical Chemistry, University Innsbruck, Innrain 52c, Innsbruck, 6020, Austria*
[‡] *Physics of Energy Conversion and Storage (ECS), Physics Department, Technical University of Munich, James-Franck-Straße 1, 85748 Garching, Germany*

[§] *Catalysis Research Center TUM, Ernst-Otto-Fischer-Straße 1, 85748 Garching, Germany*

* Corresponding author e-mail: julia.kunze@uibk.ac.at (J. Kunze-Liebhäuser),

bandarenka@ph.tum.de (A.S. Bandarenka)

[#] Contributed equally

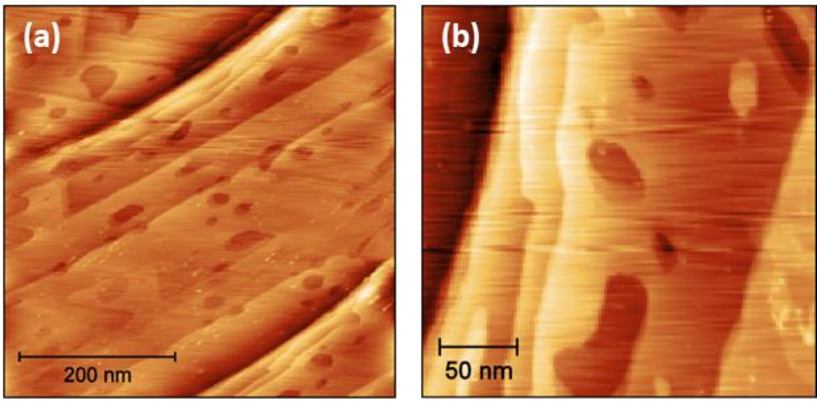

Figure S1. Electrochemical scanning tunnelling microscopy (EC-STM) images of metallic Cu(111) electrodes in Ar-saturated alkaline solution at -1.0 V_{SHE}. (a) (500 x 500) nm^2 and (b) (250 x 250) nm^2. I_{tip}= 1 nA, E_{tip}= -0.4 V_{SHE}.

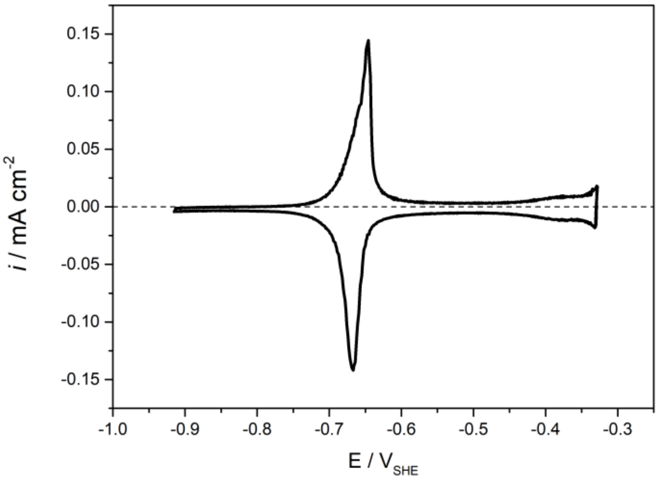

Figure S2. Reference cyclic voltammetry measurement (CV) of Cu(111) in 0.1 M NaOH (pH=13) showing the electrochemical behavior in the potential range between -0.9 V_{SHE} and -0.33 V_{SHE} (scan rate: 50 mV s^{-1}). The observed peak pair at ~ -0.65 V_{SHE} corresponds to the adsorption and desorption of OH^- ions from the alkaline solution.

Figure S2 shows a typical, state-of-the-art cyclic voltammogram of Cu(111) obtained in 0.1 M NaOH, see also ref. [1]. The peak at ~ -0.65 V_{SHE} (0.12 V_{RHE}) reflects the adsorption of OH^- anions. The charge obtained by integration of the current corresponds to an average value of around 95 µC cm^{-2}, which is in perfect agreement with the charge obtained for $NaClO_4$ (pH=13) solution, shown in Figure 2 in the main text. Therefore, it can be concluded that the ClO_4^- anions do not specifically adsorb under these highly alkaline conditions.

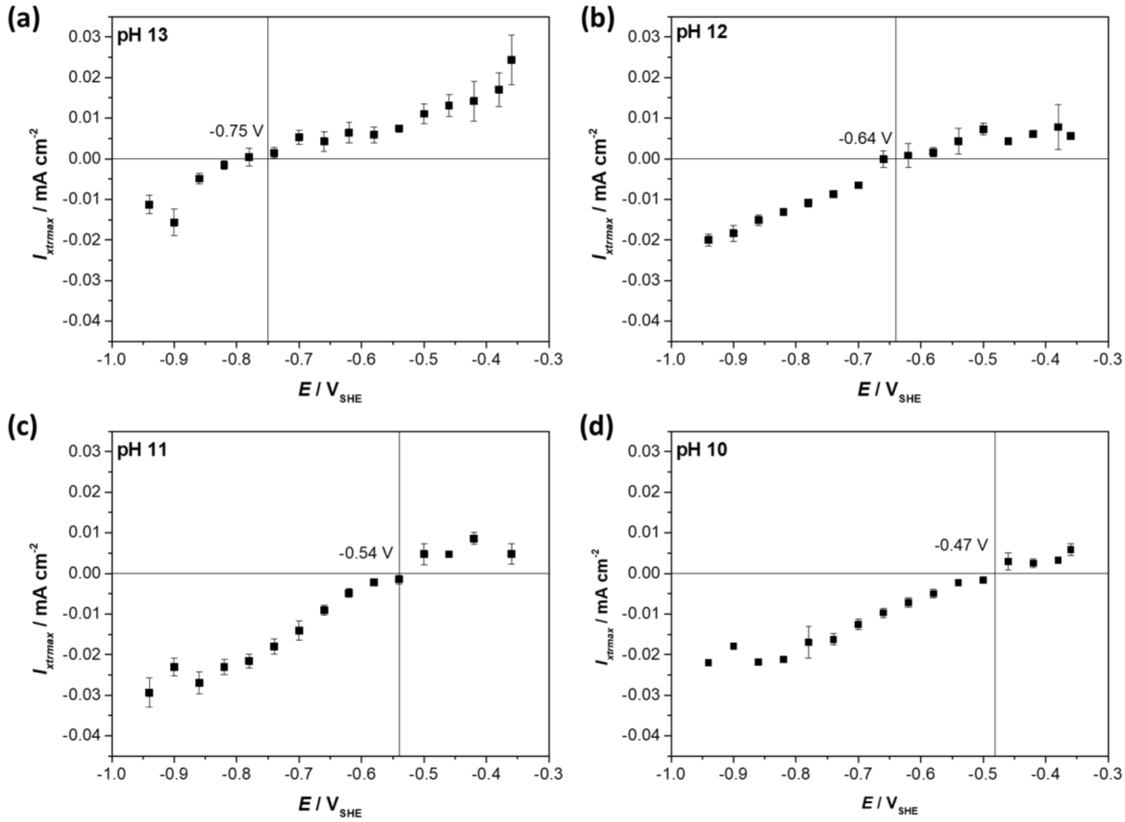

Figure S3. Maximum current values of the transients plotted versus the potential for (a) pH 13, (b) pH 12, (c) pH 11 and (d) pH 10. Mean values and standard deviations of the peak currents result from averaging three transient data sets.

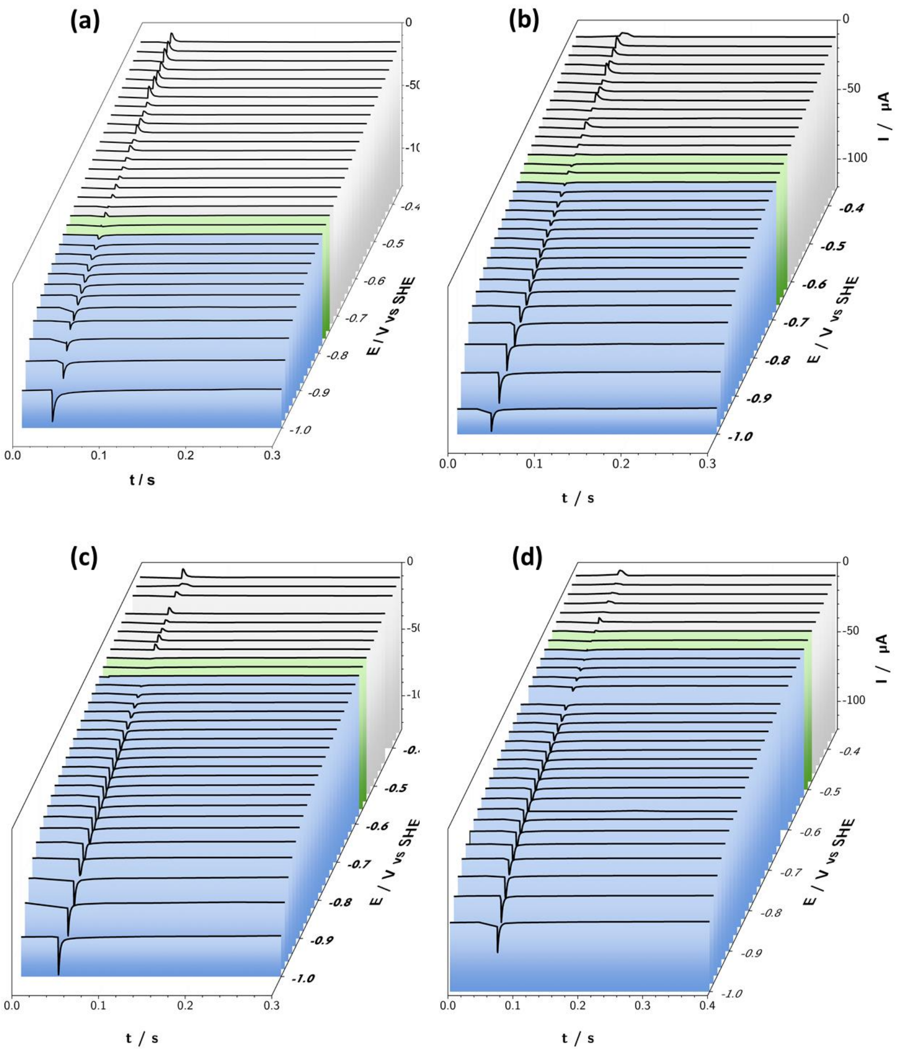

Figure S4. 3D-Plot of the laser-induced current transients of Cu(111) electrodes in $NaClO_4$ solution at (a) pH 13, (b) pH 12, (c) pH 11 and (d) pH 10. The different signs of the transients are schematically color-coded: blue corresponds to negative transients, green to the change of sign (average values and standard deviations), i.e. the potentials of zero transient, and grey to positive transients.

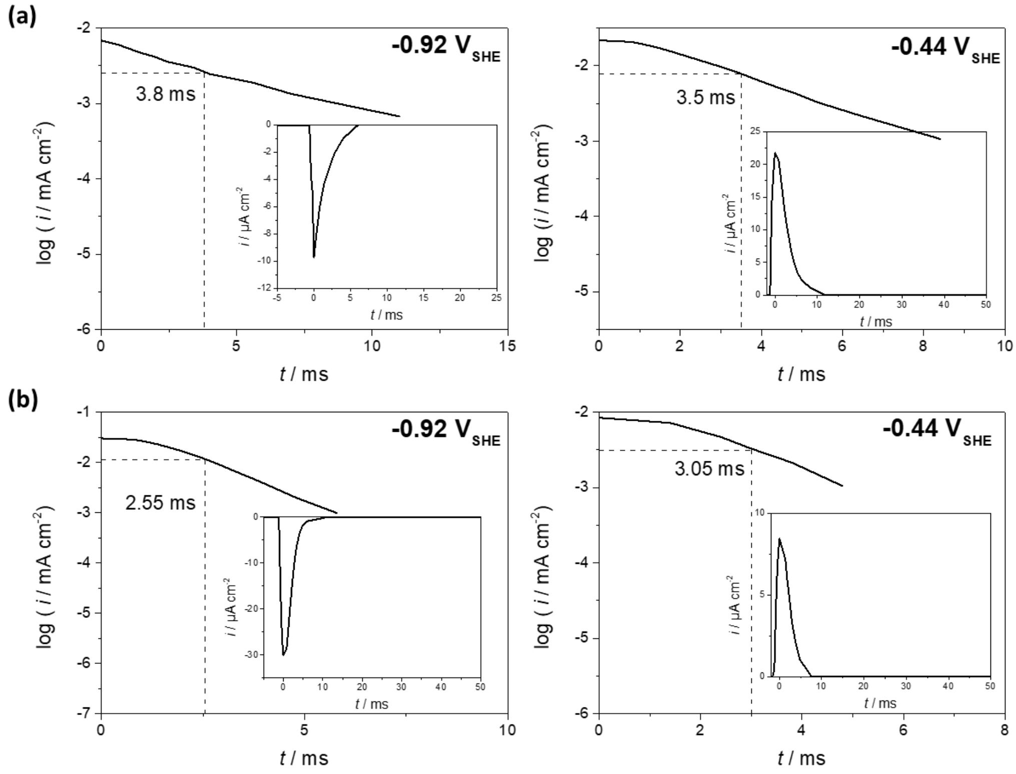

Figure S5. Semilogarithmic plots of two representative transient evolutions at two different potentials for (a) pH 13 and (b) pH 11. The insets show magnifications of the background corrected transients.

Figure S5 shows semilogarithmic plots of current transients for both pH 13 and pH 11 at two different potentials. Assuming an equivalent circuit consisting of a resistor and a capacitor in series (RC-circuit), the time constant was estimated at the position where the peak current drops to 37 % of its initial value (at t=0). Dividing the time constant by the approximate value of the double layer capacity determined from cyclic voltammetry (30 μF cm^{-2}) delivers a resistance ranging from 160- 250 Ω.

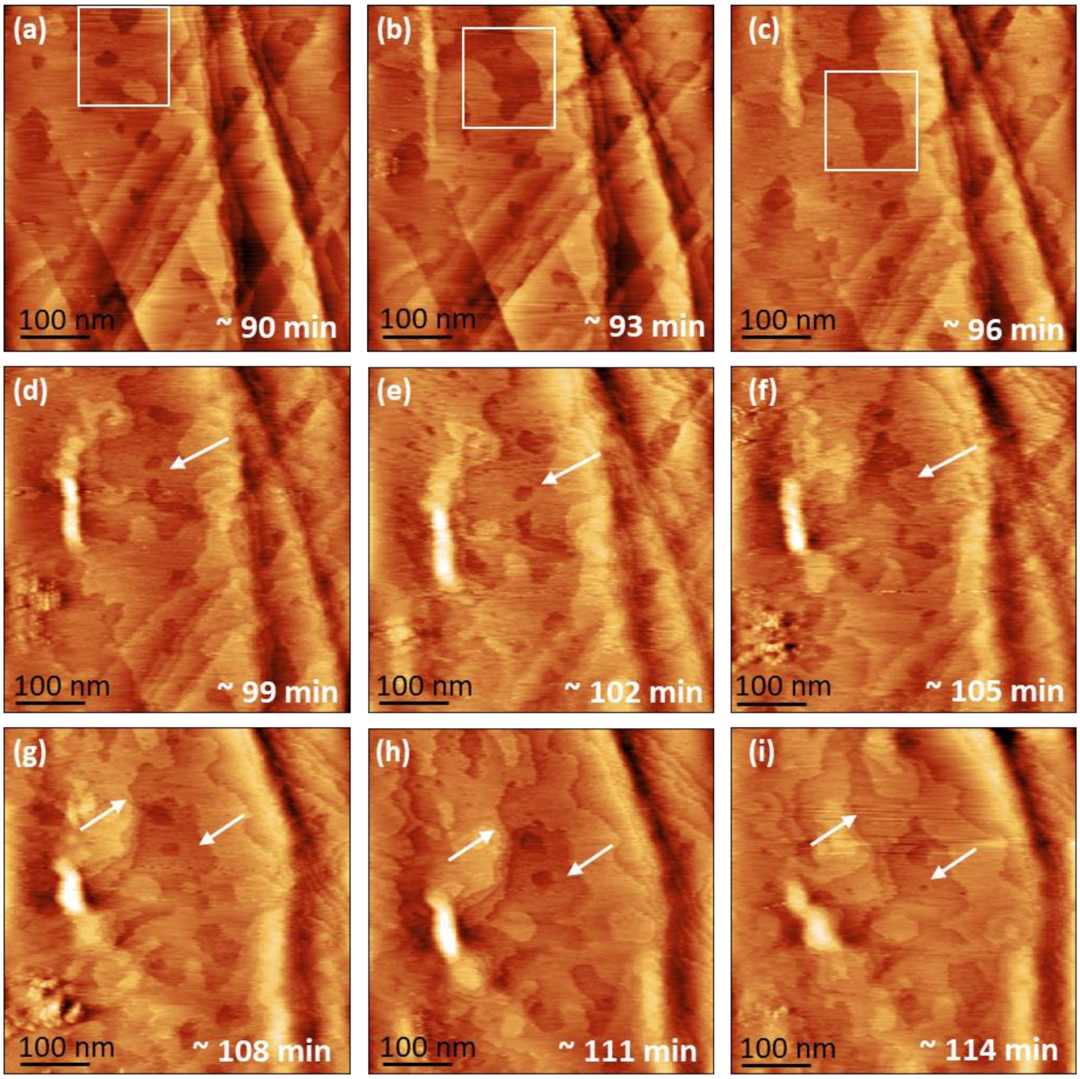

Figure S6. Sequence of low resolution EC-STM images of Cu(111) at -0.70 V_{SHE} in $NaClO_4$ (pH=13). The appearance and disappearance of (vacancy) islands are marked with squares and arrows to guide the eye ((a)-(i)). The time referred to in the images is the time elapsed since stepping to -0.7 V_{SHE}. Size = (500 x 500) nm^2, I_{tip} = 1 nA, E_{tip} = -0.50 V_{SHE}.

Figure S6 compiles a series of low resolution electrochemical scanning tunneling microscopy (EC-STM) images taken at -0.70 V_{SHE}, which is slightly positive of the determined pzfc. Compared to Figure 4 in the main text, the appearance and disappearance of (vacancy) islands through step and island coalescence can be more easily observed in these lower resolution images. The morphologies and structures imaged at -0.75 and -0.70 V_{SHE} significantly differ from those at higher, e.g. OH-adsorption potentials, where the growth of smaller Cu ad-islands is observed (Figures 5), which allows for a clear distinction between the surface structure and morphology at and close to the pzfc and upon OH-adsorption.

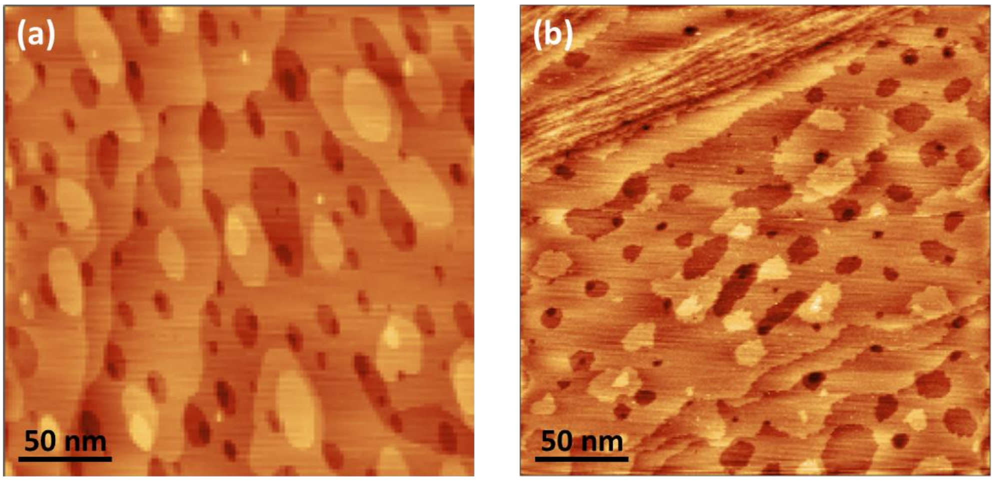

Figure S7. Comparison of the step edge morphology and structure before (a) and after (b) holding the potential at the pzfc at pH 11.

Figure S7 shows two low resolution images ((250 x 250) nm^2) to demonstrate the homogeneity of the Cu(111) surface both before and after the observed restructuring of the step edges. While at potentials negative of the pzfc smooth and even step edges are observed over the whole surface, all the step edges become rugged and rough at the pzfc.

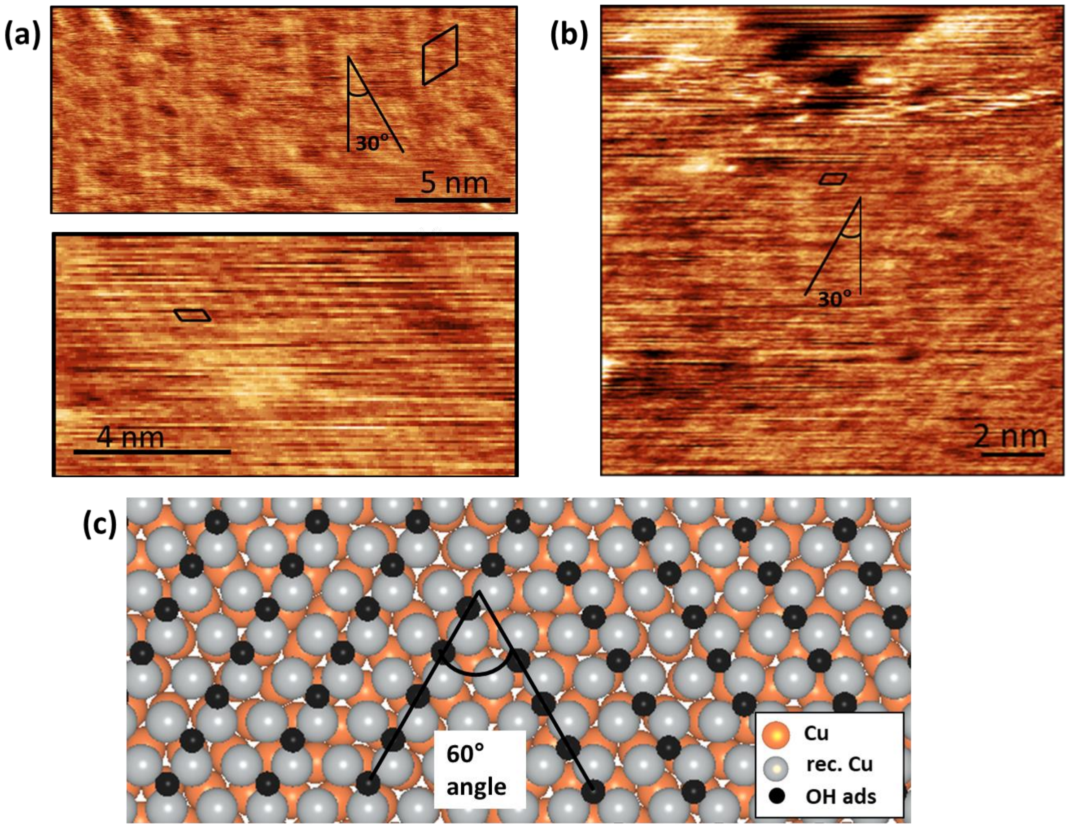

Figure S8. EC-STM images of a second adsorption domain, differently oriented with respect to the expanded Cu layer. (a)-(b) STM images showing both the Moiré and adsorbed OH-lattices in two directions. (c) Structural model of the two domains, which are rotated by an angle of 60° to each other.

Figure S8 displays high resolution images of Cu(111) in $NaClO_4$ (pH=13) at -0.55 V_{SHE}, which exhibit a short range corrugation rotated by 30° with respect to the direction of the Moiré pattern. The 30° rotation has been observed in two directions (see Figures S8 a and b) which adds up to a 60° rotational angle between the two domain directions.

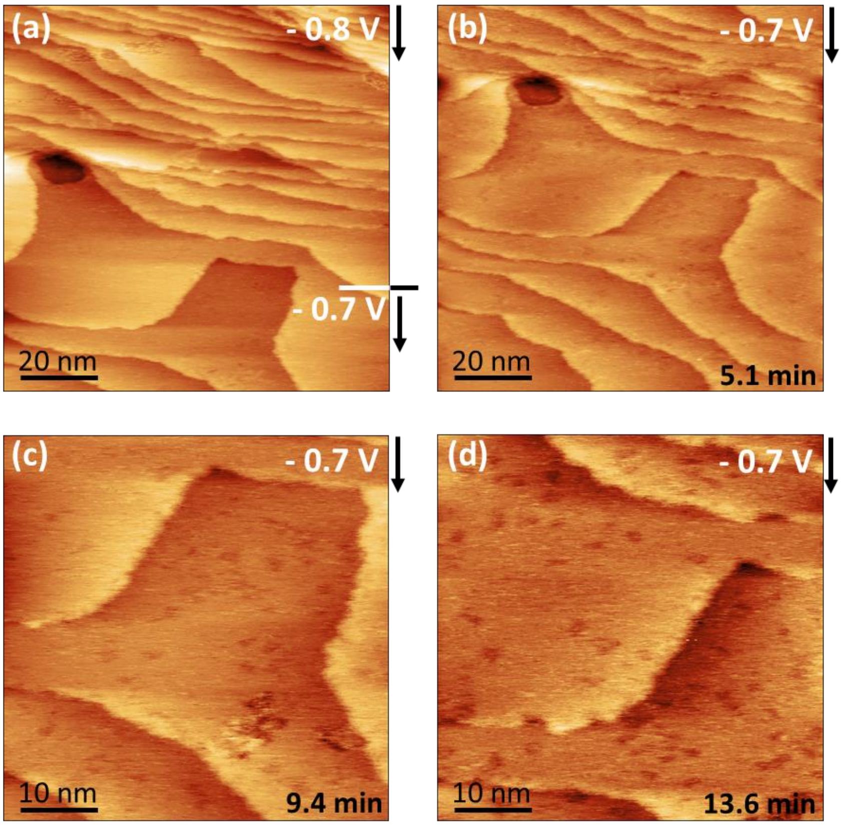

Figure S9. EC-STM images of Cu(111) in $NaClO_4$ (pH=13) during a potential step to -0.7 V_{SHE} taken in non-deaerated solution outside the glove box. (a),(b) Size = (100 x 100) nm^2, (c),(d) Size = (50 x 50) nm^2. I_{tip} = 1 nA, E_{tip} = -0.50 V_{SHE}.

EC-STM images of Cu(111) during a potential step to -0.7 V_{SHE} in non-deaerated $NaClO_4$ solution imaged outside the glove box are compiled in Figure S9. Dark spots forming on the terraces and step edges are attributed to oxygen-containing species and are clearly visible, whereas no structural changes at the steps and terraces can be observed. These results in comparison with the images shown in Figures 4 and 5, which are taken in completely oxygen-free electrolyte, clearly indicate the impact of dissolved O_2, which can adsorb and therefore pin the Cu(111) surface. The observed dark spots are furthermore well-documented and in agreement with the literature, see refs. [2,3].

Supporting References

(1) Tiwari, A.; Heenen, H. H.; Bjørnlund, A. S.; Maagaard, T.; Cho, E.; Chorkendorff, I.; Kristoffersen, H. H.; Chan, K.; Horch, S. Fingerprint Voltammograms of Copper Single Crystals under Alkaline Conditions: A Fundamental Mechanistic Analysis. *J. Phys. Chem. Lett.* **2020**, *11* (4), 1450–1455.

(2) Maurice, V.; Strehblow, H.-H.; Marcus, P. In Situ STM Study of the Initial Stages of Anodic Oxidation of Cu(111) in Aqueous Solution. *Surf. Sci.* **2000**, *458*, 185–194.

(3) Kunze-Liebhäuser, J. Electrochemical Scanning Tunneling Microscopy of Copper Oxide Formation - A Review. In *Encyclopedia of Interfacial Chemistry*; Wandelt, K., Ed.; Elsevier Inc., 2017; pp 107–120.
